# Endogenous stem cells for enhancing cognition in the diseased brain

**DOI:** 10.3389/fnins.2014.00098

**Published:** 2014-05-06

**Authors:** Angélique Bordey

**Affiliations:** Departments of Neurosurgery, and Cellular and Molecular Physiology, Yale University School of MedicineNew Haven, CT, USA

**Keywords:** neural stem cells, brain repair, subventricular zone (SVZ), mTOR, self-renewal, injury, aging, drug delivery

## Introduction

Adult neural progenitor cells or neural stem cells (NSCs) persist in the adult human brain in two well-established regions, the subventricular zone (SVZ) and the subgranular zone (SGZ) of the dentate gyrus. Newborn neurons have been observed in the human SGZ in adults and contribute to specific forms of memory encoding at least in rodents (Braun and Jessberger, 2014). Neurogenesis from the adult SVZ was primarily identified in the olfactory bulb in rodents and was shown to stop early in life in humans despite the continuous presence of NSCs (Sanai et al., 2011). However, a recent study reports neurogenesis in the striatum from the adult human SVZ (Ernst et al., 2014). This finding highlights the difference between rodents and humans and the fact that some brain regions display an unexpected capacity for newborn neuron migration and survival. The SVZ is a prime region to consider for brain repair considering that it spans the entire cerebrum while the SGZ is limited to the hippocampus. Other regions are now known to contain NSCs or progenitor cells such as the hypothalamus, but this will not be discussed here. Several milestones need to be achieved prior to considering functional repair. These include, but may not be limited to: (1) Understanding the mechanisms leading to NSC quiescence and loss with aging. Several mechanisms are involved in the different regulatory steps of NSC self-renewal and loss. We will emphasize some of the mechanisms leading to NSC loss with aging. Once these mechanisms are identified, we should be able to amplify the pool of NSCs and direct their differentiation. (2) Identifying the molecules responsible for fate determination of NSCs and their daughter cells to generate glia or neurons of different types, including interneurons and long projection neurons. (3) Determining the inhibitory molecules that make the adult brain resistant to repair. Some repair has been reported in the cortex of rodents, but it is abortive possibly due to an unfriendly environment. (4) Finally, although we can genetically manipulate NSCs in rodents, it is a different issue in humans. Delivery systems need to be improved. Each of this point is further discussed below.

## Understanding the mechanisms leading to adult NSC quiescence and loss with aging

Significant amount of work has been focused on understanding the signaling molecules and pathways involved in the regulation of NSC quiescence (Basak and Taylor, 2009). For example, two major players are the Notch and Wnt signaling pathways. Most of the identified pathways are conserved between embryonic and adult NSCs although some molecular players may differ. They are also conserved across NSC niches including in the periphery (Fuchs et al., 2004). Although a large repertoire of molecules have been identified, some confusion remains regarding the true molecular identity of the dormant, quiescent and activated (i.e., proliferative) NSCs. The question why a dormant NSC becomes activated remains unanswered. In others terms, what are the molecules necessary and sufficient to wake up NSCs? Whether these molecules are the same for all NSCs remain unclear.

In addition, an injury to the brain may dramatically affect the activation state of molecular pathways in NSCs as well as their microenvironment. Very little is known on how NSCs respond to injury in terms of their molecular signature driving them out of dormancy. To make matter more complicated, every injury may not alter NSCs in similar fashion. These questions need to be fully examined.

Aging is a natural phenomenon affecting NSCs and their microenvironment (van and Franklin, 2013). One clear outcome of aging is a loss of NSCs and thus reduction in neurogenesis. The extent and mechanisms of NSC loss are likely different in the SGZ and NSC (Shruster et al., 2010). In the SGZ, NSCs terminally differentiate into astrocytes (Encinas et al., 2011). In the SVZ, there is a progressive loss of transit amplifying cells with aging (Paliouras et al., 2012). At the molecular level, one key player in aging is the mammalian target of rapamycin complex 1 (mTORC1) pathway, which controls cap-dependent protein translation (Johnson et al., 2013). There are no data for mTORC1 contribution to aging in the SGZ. In the SVZ, mTORC1 has been involved in the loss of NSC with aging (Paliouras et al., 2012). In addition, activation of this pathway during aging has been proposed to be involved in the terminal differentiation of NSCs into daughter cells, thus contributing to NSC loss (Hartman et al., 2013). Thus, whether small amount of the mTORC1 blocker, rapamycin, could prevent progressive NSC loss is to be examined. mTORC1 activity increases with aging in other systems and rapamycin has been shown to prolong life of animals.

## Identifying the molecular mechanisms controlling fate determination to generate every neuron type

Adult NSCs essentially generate neurons, most specifically interneurons. In the SVZ, they were shown to be genetically predetermined to generate specific subclasses of olfactory interneurons (Merkle et al., 2007). The genetic network involved in this specificity remains to be identified. In addition, identifying the molecular program determining this fate restricted genetic network needs to be examined earlier during development. In other terms, we will learn from studies focused on the identification of the programs at play in embryonic NSCs that determine their restricted fate once adult NSCs.

With brain repair in mind, there is a need to generate long range projection neurons and not just interneurons. Progress needs to be made in our abilities to reprogram adult NSCs to resemble embryonic NSCs with broader fate determination. Novel reprogramming technologies are developed exponentially in line with the discovery of induced pluripotent stem cells. This technologies need to be applied to adult NSCs *in vitro* and *in vivo*.

## Determining the inhibitory molecules making the adult brain resistant to repair

Perhaps as a protective mechanism for maintaining long-term memory, the adult brain displays very limited repair despite the presence of NSCs in the human SVZ and the reported existence of cells with neural progenitor property in the parenchyma. The adult brain contains repulsive cues (e.g., NOGO and similar factors) and lack nurturing cues for NSC and newborn neurons to survive and integrate, thus providing a non-permissive environment for neurogenesis. There is a definite need for studies aimed at identifying the repulsive cues present in the adult parenchyma preventing NSC and immature neuron survival and integration in normal and injury conditions. In addition, it will be important to compare the molecular signature of different brain regions that display different permissiveness to repair such as the striatum versus the cortex (Ernst et al., 2014). Large molecular screens as well as hypothesis-driven approaches could be taken to address this issue.

One remaining major limitation is access to human brain tissue. The human brain is likely more resistant to repair than the mouse brain. Efforts to develop approaches to perform screen in human tissue and identify factors preventing endogenous neurogenesis need to be developed.

## *In vivo* therapeutic manipulations

Even if we address all of the issues listed above, we are left with the task of developing strategies to manipulate cells in the human brains in the least invasive way possible. So far, the best strategy has been to use pharmacological treatments in humans. However, reprogramming NSCs to generate new types of neurons will require transferring DNA or genetic material into cells. In the rodent brain, such strategy is achieved in specific cell types using transgenic animals, viral delivery, electroporation, nanoparticle or exosomal delivery systems, and to some extent cell-penetrating peptides. Nanoparticle and exosomal strategies are promising for humans. In particular, non-invasive intra-nasal delivery is attractive and should be further explored. For success in developing *in vivo* delivery systems in humans, biologists and bioengineers will need to exchange concepts and work together.

## Conclusion

**Figure 1:**
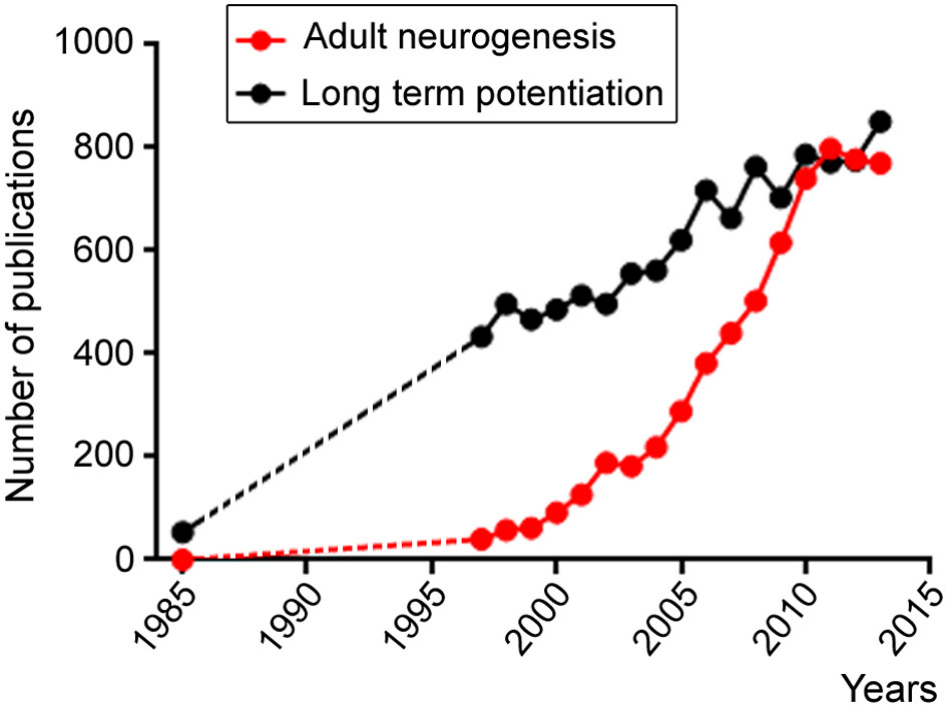
**Scattered plots illustrating the number of publications per year obtained in Pubmed using the following two key words: adult neurogenesis (red) and long term potentiation**. Both fields display increases over time, but the adult neurogenesis field displays an exponential increase starting in the late 1990's while the long term potential field displays a linear increase likely reflecting the increase in the number of scientists during this period.

Despite the hurdles outlined above and the length of time that will be required for achieving brain repair and cognitive enhancement, we cannot fail to pursue our investigations of the four fields outlined above. In the past decade, there has been an exponential increase in studies related to the field of “adult neurogenesis.” This is outlined in Figure 1 following a search in PubMed with this keyword compared to a search with “long term potentiation,” which outlines the growth of the neuroscience field. Although there is an apparent stabilization of the growth of the adult neurogenesis field since 2011, this likely reflects the expansion of the field and thus the need to use additional keywords in our search such as adult NSC, repair in the adult brain, adult SVZ or SGZ. This is my hope and belief that despite economic recession affecting scientific growth the number of labs studying adult neurogenesis and NSCs is still increasing.

Overall, the present energetic study of stem cell biology and brain delivery systems will provide a better understanding of brain development, endogenous responses to injuries, and additional therapeutic approaches for brain repair, and hold great promise for broadening the therapeutic options available for maintaining and restoring cognition following brain injury and during neurodegenerative diseases. Finally, validating that findings obtained in animal models apply to humans is a must. While some molecules may be the same, the pathways may be different or act differently and this needs to be validated in human adult NSCs prior to trying to repair the human brain.

### Conflict of interest statement

The author declares that the research was conducted in the absence of any commercial or financial relationships that could be construed as a potential conflict of interest.
